# HOW DO INTERPERSONAL FACTORS AFFECT GRIEF AMONG BLACK MIDDLE- TO OLDER-AGED ADULTS?

**DOI:** 10.1093/geroni/igac059.246

**Published:** 2022-12-20

**Authors:** Danielle McDuffie

**Affiliations:** The University of Alabama/ Durham VAMC, Tuscaloosa, Alabama, United States

## Abstract

This talk will explore: 1) the conceptual importance of select interpersonal factors inherent to Black culture and bereavement, and 2) the impact of these factors on grief in a Black bereaved sample. 103 Black adults aged 45+ were administered items assessing cultural (communalism, experienced racial violence) and bereavement-specific (expectancy and suddenness of loss, closeness to the deceased, anger) factors. Using linear regressions, closeness (p<.001), suddenness (p=.020), anger (p<.001), and communalism (p<.001) predicted reported levels of grief. This study identifies factors which substantially dictate the experienced levels of grief of bereaved Black adults in the latter half of life. With cultural factors notably impacting the Black bereavement experience, individuals seeking to work with this population must adopt strategies to keep bereaved Black adults connected to their communities throughout their loss. Resources to support Black adults in maintaining healthy continuing bonds with the deceased through abrupt or unsettling losses also appear paramount.

